# Bone Regeneration Induced by Bone Porcine Block with Bone Marrow Stromal Stem Cells in a Minipig Model of Mandibular “Critical Size” Defect

**DOI:** 10.1155/2017/9082869

**Published:** 2017-05-02

**Authors:** Antonio Scarano, Vito Crincoli, Adriana Di Benedetto, Valerio Cozzolino, Felice Lorusso, Michele Podaliri Vulpiani, Maria Grano, Zamira Kalemaj, Giorgio Mori, Felice Roberto Grassi

**Affiliations:** ^1^Department of Medical, Oral and Biotechnological Sciences and CeSI-Met, University of Chieti-Pescara, Chieti, Italy; ^2^Interdisciplinary Department of Medicine, University of Bari "A. Moro", Bari, Italy; ^3^Department of Clinical and Experimental Medicine, Medical School, University of Foggia, Foggia, Italy; ^4^Department of Animals Sperimentation, Istituto Zooprofilattico Sperimentale dell'Abruzzo e del Molise "G. Caporale", Teramo, Italy; ^5^Department of Emergency and Organ Transplantation, University of Bari "A. Moro", Bari, Italy; ^6^Department of Surgical Sciences, Dental School, University of Turin, Torino, Italy; ^7^Department of Basic and Medical Sciences, Neurosciences and Sense Organs, University of Bari, Bari, Italy

## Abstract

*Introduction*. Adding stem cells to biodegradable scaffolds to enhance bone regeneration is a valuable option. Different kinds of stem cells with osteoblastic activity were tested, such as bone marrow stromal stem cells (BMSSCs). *Aim*. To assess a correct protocol for osteogenic stem cell differentiation, so BMSSCs were seeded on a bone porcine block (BPB). *Materials and Methods*. Bone marrow from six minipigs was extracted from tibiae and humeri and treated to isolate BMSSCs. After seeding on BPB, critical-size defects were created on each mandible of the minipigs and implanted with BPB and BPB/BMSSCs. After three months, histomorphometric analysis was performed. *Results*. Histomorphometric analysis provided percentages of the three groups. Tissues present in control defects were 23 ± 2% lamellar bone, 28 ± 1% woven bone, and 56 ± 4% marrow spaces; in BPB defects were 20 ± 5% BPB, 32 ± 2% lamellar bone, 24 ± 1% woven bone, and 28 ± 2% marrow spaces; in BPB/BMSSCs defects were 17 ± 4% BPB/BMSSCs, 42 ± 2% lamellar bone, 12 ± 1% woven bone, and 22 ± 3% marrow spaces. *Conclusion*. BPB used as a scaffold to induce bone regeneration may benefit from the addition of BDPSCs in the tissue-engineered constructs.

## 1. Introduction

### 1.1. Bone Regeneration with Stem Cells

A common problem facing the dental community is the rehabilitation of oral functions in patients with edentulous atrophic alveolar process. Improvement of successful methods to induce bone regeneration is a continuous challenge in dentistry [1]. In recent years, the use of biomaterials to enhance bone regeneration has greatly developed because of their capacity to mimic the natural environment of the extracellular matrix [2]. To increase the effectiveness of this methodology, the use of biomaterials, or scaffolds, in association with stem cells with osteoblast-like activity has been introduced [3, 4]. In 1968, Friedenstein et al. first published works demonstrating the osteogenic property of bone marrow transplants in different tissues and the possibility of cultivating, cloning, and retransplanting in vivo [5]. A number of stem cell studies have followed [6, 7]. Adult mesenchymal stem cells can be obtained from many sources. In particular, they can successfully be differentiated in osteoblast-like cells originating from different dental tissues [8–11].

### 1.2. BMSSC

Among the stem cells with the desired osteoblastic activity, BMSSCs have proved effective in inducing new bone formation in critical-size defects of animal models [12–14]. Indeed, the ability of those cells to enhance bone formation can even be found in scaffold-type constructions, tridimensional shapes, and the culturalization of cells. In recent years, several matrices have been used, from nonresorbable biomaterials, such as hydroxyapatite, in different relationships with BMSSCs, including layers encapsulated in hydrogel [15] or calcined bovine bone [16], to resorbable ones such as beta-tri-calcium phosphate (*β*-TCP) or calcium phosphate (CP) [17]. When comparing matrices, one study found a better response in vivo and in vitro from calcium phosphate rather than hydroxyapatite on increased trabecula formation, cell density, and decreased fibrosis [18]. Regardless of the results, there is still no universally accepted scaffold and each one has to be tested individually.

On those scaffolds, BMSSCs are cultured in situ improving bone healing and matrix reabsorption with an osteogenic effect. In a recent experiment, BMSSCs with an enriched chitosan scaffold produced bone, more than CP alone in rat muscle [19]. BMSSCs are the nonhematopoietic elements of the bone marrow (BM). This cluster of cells comprises less than 0.01% of the overall cell population residing in the BM [20]. The nonhomogeneous nature of BMSSCs is clearly evident when examining individual colonies. Different cell morphologies include spindle cells, fibroblast-like cells, or colonies of large and flat-shaped cells. Furthermore, if such cultures are allowed to develop for up to 20 days, phenotypic heterogeneity is also noted. Some colonies are highly positive for alkaline phosphatase (ALP), while others are negative, and a third type is positive in the central region and negative in the periphery [21]. Some colonies form nodules of mineralized matrix which can be identified by alizarin red or von Kossa staining for calcium. Yet others accumulate fat, identified by oil red O staining [22], while some colonies form cartilage as identified by alcian blue staining [23]. In a recent study [24] on mice mesenchymal stem cells derived from dental pulp and periosteum, a difference in bone regeneration was found, confirming the hypothesis of enhancing regeneration with mesenchymal stem cells. It was measured as a percentage of bone volume on the total defect area, when seeded with scaffold block deproteinized porcine bone (BDPB) alone and with dental pulp and periosteal stem cells. Qualitative observations on bone histomorphometry classify the nature of bone elements as either lamellar or woven. The presence of residual biomaterial and inflammatory mediators within the defects was also observed. A similar model has been adopted in this study to evaluate different degrees of bone regeneration using bone porcine block with bone marrow stromal stem cells (BPB/BMSSCs).

The aim of this study is to assess a correct protocol for osteogenic stem cell differentiation of an osteoblastic phenotype on an appropriate substrate: BMSSCs seeded on a bone porcine block (BPB) in order to increase performance in bone regeneration using scaffold from the block and inducing a qualitative superior regeneration with BMSSCs.

## 2. Materials and Methods

### 2.1. Animals

Six adult minipigs of a mean age of 2 years were used in the present study. The mean of weight was 29 kg ± 4 kg. The study protocol was approved by the Italian National Health (protocol number 7326-26/06/2013).

The animals were maintained according to the guidelines for ethical conduct developed by the European Communities Council Directive of November 24, 1986 (86/609/EEC). All efforts were made to minimize pain or discomfort of the animals. In each emi-mandibula, 3 defects (5 mm wide and 5 mm deep) were created. The defects were then filled in the following way: one defect with a bone porcine block (BPB) (OsteoBiol, Tecnoss, Coazze, Italy) with BMSSCs was inserted, one filled with bone porcine block left without BMSSCs empty, and one defect was left blank as control. A total of 36 critical-size circular defects (5 mm diameter; 5 mm thickness) were created.

### 2.2. In Vitro Cell Culture

Bone marrow was harvested according to the following surgical technique.

In the minipigs, the bone marrow was harvested from the proximal tibiae and humeri. The volume of blood circulating in pigs is 65–75 ml/kg. The animals used for the experiments were about ~30 kg of body weight; thus, a total volume of 20 ml of bone marrow per animal was safely collected, specifically 5 ml per bone segment considering the two humeri and the two tibias, obtaining approximately 2 × 10^9^ bone marrow mononuclear cells (BMMNCs) per pig [25–27]. The pigs were anesthetized as previously described. The technique was performed in an aseptic environment and using sterile gloves and instruments, after shaving and a thorough disinfection of the skin in the harvesting site. The procedure was realized with a needle specifically built to harvest the bone marrow, 11 gauges × 110 mm, which has a cutting edge and a spindle. The harvesting site was in correspondence with the medial tibia and the proximal humerus of both limbs, where the compact bone is more compliant having this area a relatively thin cortical bone. The pigs were placed in the lateral decubitus position. The bone marrow aspiration needle was passed through the skin with the spindle in position. In the the cortical bone, the spindle was removed, and the bone marrow, in the volume of 5 ml for skeletal segment, was aspirated into a sterile syringe containing 0.2 ml of heparin (1000 units/ml). The samples were quickly transferred to the laboratory for the subsequent steps of isolation and stem cell differentiation.

### 2.3. BMSSC Isolation, Culture, and Scaffold Preparation

**Figure 1:**
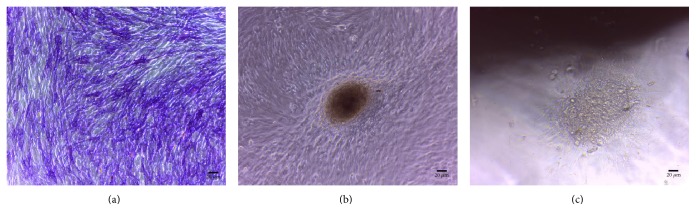
(a) In vitro appearance of BMSSCs forming a matrix nodule after two weeks of culture in the presence of osteoblast differentiation medium. (b) Cultures of BMSSCs stained for ALP expression. (c) In vitro appearance of BMSSCs forming a matrix nodule in proximity of BPB before grafting.

In order to obtain BMSSCs, bone marrow aspirates were processed as previously described briefly [28, 29]: samples were subjected to Histopaque 1077 density gradient (Sigma-Aldrich, St. Louis, MO, USA). The buffy coat cell fraction was entirely harvested and incubated in a humidified atmosphere of 5% CO2 at 37°C to obtain the BMNCs: after 24 hours, the nonadherent cell fraction was discarded while the adherent cell fraction was cultured for 48–72 hours until it reached a semiconfluent status (Figure 1(a)).

The BMSSC cultures, thus obtained, were trypsinized, counted, and used to be tested and then integrated in the scaffolds. To control the osteogenic differentiation, a cell fraction was cultured in the presence of ascorbic acid 50 ng/ml for ten days and then stained for alkaline phosphatase (ALP) expression as previously described (Figure 1(b)). From each animal, 1 × 10^6^ BMSSCs were used for flow cytometric assay that demonstrated mesenchymal stem cell marker expression. The bone porcine block scaffold used presented a cylindrical shape, 5 mm in diameter, and 5 mm in length. They were rigid cancellous blocks and thus are able to maintain in time the original graft volume, which is particularly important in cases of large regenerations.

Before being integrated with the cells, the scaffolds were hydrated in alpha-MEM and incubated three times for 30 min in a humidified atmosphere of 5% CO2 at 37°C. Prior to cell seeding, the volume of medium contained in the tridimensional structure of the scaffolds was removed by absorption using sterile cotton balls. Then 100 *μ*l BMSSC suspension (5 × 10^5^ cells) was slowly dripped onto the scaffolds to avoid overflow. The scaffolds seeded with BMSSCs were incubated in a humidified atmosphere of 5% CO2 at 37°C for 2 h, after which the additional culture medium was added to fully cover the scaffolds. To ensure that cells can successfully attach to the scaffolds, the cultures were incubated in a humidified atmosphere of 5% CO2 at 37°C for 3 days. After 3 days, 50 *μ*g/ml ascorbic acid was added and the media was changed every 2 days for two weeks in order to obtain, before scaffold grafting, BMSSC osteogenic differentiation on the cells integrated into the scaffolds (Figure 1(c)).

### 2.4. In Vivo Mandibular Defect

**Figure 2:**
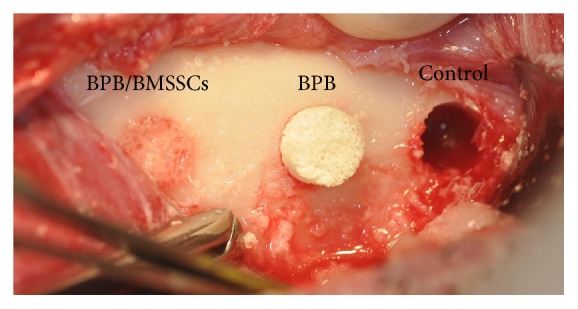
Three critical-size circular defects. Clinical situation during surgery.

Surgery was performed under general anesthesia with induction of Zoletil 100 (tiletamine hydrochloride + zolazepam hydrochloride) at a dosage of 6 mg/kg IM. Maintenance with isoflurane at 2/2.5% in oxygen. Three bilateral critical-size circular defects (5 mm diameter; 5 mm thickness) were created using a hand drill and trephine bit in the mandibular body (Figure 2). Posterior region of the mandible was chosen because of the presence of regular thickness of the vestibular cortical plate; defects were created with those specific measures to better place the cylindrical bone porcine blocks, perfectly adapted, into the defects. Each defect was filled randomly by the surgeon. During the procedure, sterile saline was dripped over the drilling site in order to avoid extensive heating and to protect surrounding bone.

Treatment of postoperative pain with flunifen (flunixin meglumine) at a dosage of 2.2 mg/kg IM once (for all) or twice depending on whether or not the animal showed pain. Antibiotic treatment with Repen (Benzilpenicillina dihydrostreptomycin) at a dosage of 20.000 IU of Benzilpenicillina and 12.5 mg of dihydrostreptomycin/kg for 5 days IM.

### 2.5. Histomorphometry

**Figure 3:**
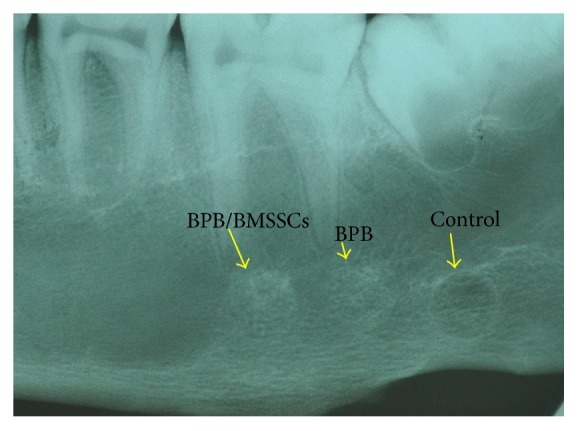
Standard RX 55 × 75 mm films have been performed on retrieved mandibulae to find the perfect position and alignment of biomaterial scaffolds with the bone itself.

The specimens were washed in saline solution and immediately fixed in 4% paraformaldehyde and 0.1% glutaraldehyde in 0.15 M cacodylate buffer at 4°C and pH 7.4, to be processed for histology. Cone beam computed tomography (CBCT) (Vatech Ipax 3D PCH-6500, Fort Lee, NJ USA) and standard RX 55 × 75 mm films (Figure 3) were performed on retrieved mandibulae. DICOM data were elaborated with Ez3D Plus Software (EZ3D Plus, VATECH Global Fort Lee, NJ USA) to elaborate 3D model specimens and find the perfect position and alignment of biomaterial scaffolds with the bone itself. CBCT analysis also permits to evaluate interface between the scaffold and the surrounding native bone. After scaffold position identification by CBCT, the posterior region of mandibulae was processed with a band saw to obtain thin ground sections with the Precise Automated System (Assing, Roma, Italy) The specimens were dehydrated in an ascending series of alcohol rinses and embedded in a glycolmethacrylate resin (Technovit 7200 VLC, Kulzer, Wehrheim, Germany). After polymerization, the specimens were sectioned in mesiodistal direction with 50 *μ*m of distance from one slide to the subsequent one, with a high-precision diamond disc at about 150 *μ*m and ground down to about 30 *μ*m with a specially designed grinding machine. A total of 3 slides were obtained for each specimen. The slides were stained with acid fuchsin and toluidine blue, and they were observed in normal transmitted light under a Leitz Laborlux microscope (Leitz, Wetzlar, Germany).

Histomorphometry was carried out using a light microscope (Laborlux S, Leitz, Wetzlar, Germany) connected to a high-resolution video camera (3CCD, JVC KY-F55B, JVC®, Santa Clara, CA, USA) and interfaced to a monitor and PC (Intel Pentium III 1200 MMX, Intel®, Yokohama, Japan). This optical system was associated with a digitizing pad (Matrix Vision GmbH, Oppenweiler, Germany), and a histometry software package with image capturing capabilities (Image-Pro Plus 4.5, Media Cybernetics Inc., Immagini & Computer Snc Milano, Italy). Evaluation of the percentages of residual biomaterial, new bone formation, and marrow space was performed in the mandibular defects of the three experimental groups. Each section was examined at at least 6x magnification, and the entire area of the section was evaluated. Digital images of each section were acquired and used to trace the areas identified as new bone, residual particle, and marrow spaces. Image manipulation software was used to create individual layers of new bone, residual particles, and marrow spaces. These layers were then converted to a binary (black and white) form, and the area by percentage of each of the three layers was digitally calculated, based on the number of pixels, using image analysis software.

### 2.6. Statistical Analysis

A power analysis was performed using clinical software, freely available on the site http://clincalc.com/stats/samplesize.aspx, for determining the number of bone defects needed to achieve statistical significance for quantitative analyses of histomorphometry. A calculation model was adopted for dichotomous variables (yes/no effect) by putting the effect incidence designed to caution the reasons at 20% for controls and 95% for treated. The optimal number of bone defects for analysis is 10, whereas in the present study, 12 defects were created per group.

Analysis of variance was used to determine the statistical significance of the differences between the three examined groups. The percentages of new bone formation, marrow spaces, and residual biomaterial were expressed as means ± standard deviations. The significance of the differences observed was evaluated using the Bonferroni test for multiple comparisons: threshold for statistical significance was set at *P* < .05.

## 3. Results

Histomorphometric results after analysis showed difference in bone regeneration between the BPB and BPB/BMSSC groups evaluated measuring percentages of the lamellar bone, woven bone, marrow spaces, and residual BPB. The control group showed a regular healing pattern. No macroscopic defects were found after three months.

### 3.1. Controls

**Figure 4:**
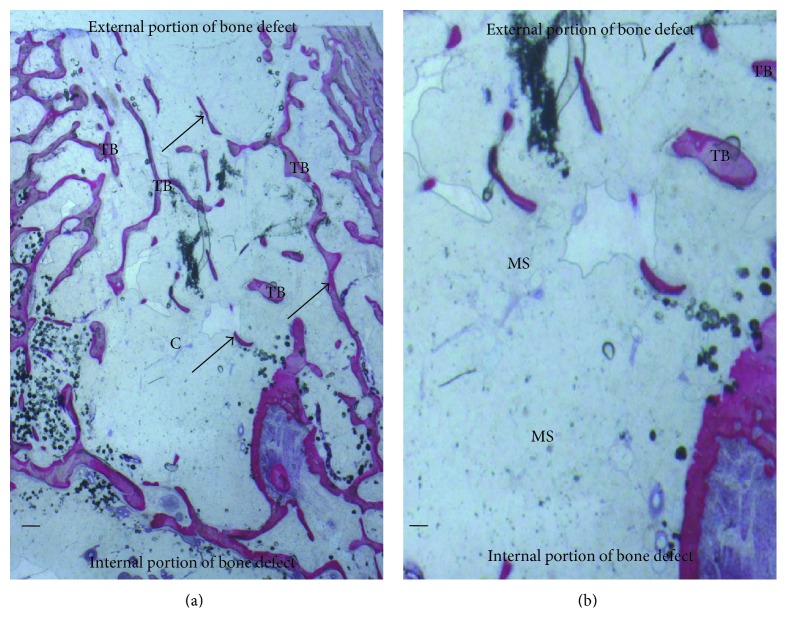
(a) Control group. Trabecular bone (TB) was present in the central portion of the bone defects (c). New bone (arrows) extended to the basal third from the margin of the bone defect. Acid fuchsin and toluidine blue. Bar = 200 *μ*. (b) Previous image at higher magnification. Isolated trabecular bone (TB was seen throughout the medullary space (MS). Acid fuchsin and toluidine blue. Bar = 200 *μ*.

Bone tissue was evident with well-differentiated cells and mineralized matrix: osteoid, osteoblasts, osteocytes, and blood vessels. Regenerating osseous tissue extending from the margin of the axial walls was observed. Fibrous tissue still occupied little space in the defect area (Figure 4). Traces of new bone from the periosteum were observed, overlying the superficial portion of the bone defect. The tissues present in the defect were composed of 23 ± 2% of the lamellar bone, 28 ± 1% of the woven bone, and 56 ± 4% of the marrow spaces.

### 3.2. Bone Porcine Block (BPB)

**Figure 5:**
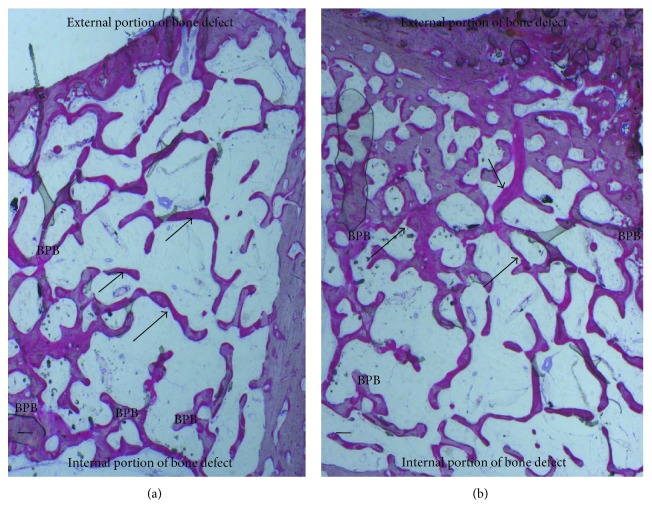
(a) BPB group. New bone surrounded the block material (BPB). New bone (arrows) extended also in the central part of the bone defects. Acid fuchsin and toluidine. Bar = 200 *μ*. (b) BPB/BMSSCs group. New bone (arrows) was deposited in the block material (BPB). No fibrous tissue was observed in the defect area. Acid fuchsin and toluidine. Bar = 200 *μ*.

**Figure 6:**
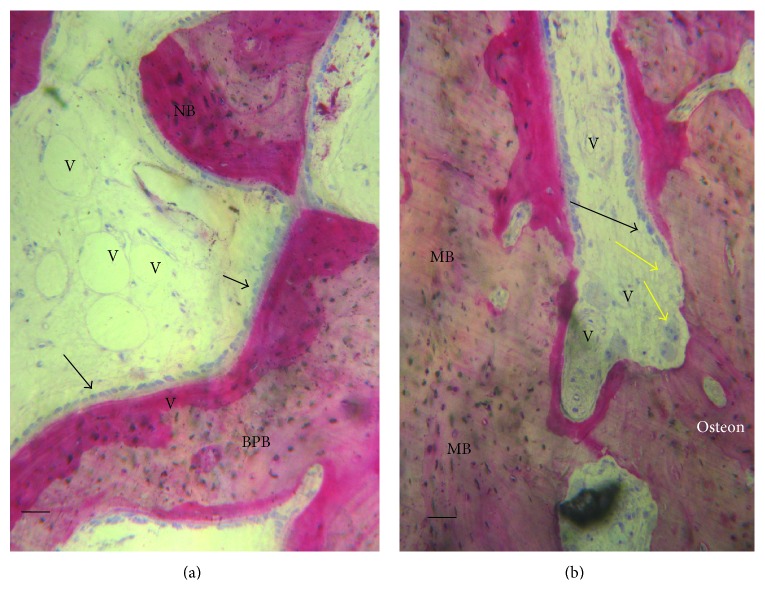
(a) BPB group. Bone tissue was deposited in the block material (BPB). No fibrous tissue was observed in the defect area. Vessels (V) were present in the central part of the bone defects. New bone (NB) and osteoblasts in close contact with the block material acid fuchsin and toluidine blue. Bar = 100 *μ*. (b) BPB/BMSSCs group. Mature bone (MB) in the bone defect. Bone morphology was more mature and well organized, presenting a primary osteon. A basic multicellular unit of osteoclasts cells (yellow arrows) that dissolves an area of the bone surface and then fills it with new bone by osteoblasts (white arrows) to form haversian systems or osteons. A vessels (V) were present in the central part of the bone defects. Acid fuchsin and toluidine blue. Bar = 100 *μ.*

At low magnification, it was possible to observe that almost all the block materials were surrounded by the mature bone: only around some fields was it possible to observe the presence of the osteoid material. The material particles were near marrow spaces in only a few areas. In all specimens, no acute inflammatory cell infiltrate or foreign body reactions were present around the particles or at the interface with the bone. All blocks were colonized and surrounded by the newly formed bone (Figure 5(a)). This bone was woven or lamellar. Inside the BPB, there was always the presence of newly formed bone. No epithelial cells or connective tissues were observed at the interface. The regenerated bone tissue extended to approximately all of the bone defects except in the central area, where the fibrous tissue still occupied a part of the interparticular spaces. Prominent lamination of the mature bone was observed. The periphery and central portion of the experimental cavities showed mineralized new bone formation (Figure 6(a)). The bone defects were not completely healed, and many particles or BPB were visible. The tissues present in the defects were composed of 20 ± 5% of BPB, by 32 ± 2% of the lamellar bone, 24 ± 1% of the woven bone, and by 28 ± 2% of the marrow spaces.

### 3.3. Bone Porcine Block with Bone Marrow Stromal Stem Cells (BPB/BMSSCs)

Regenerating osseous tissue was observed surrounding some BPB extending from the margin of the axial walls. Bone tissue was deposited in the block (Figure 5(b)). New bone extended to the basal third from the margin of the bone defect and partially surrounded the BPB block. New bone extended also in the central part of the bone defects (Figure 5(b)). No fibrous tissue was observed in the defect area. Traces of new bone from the periosteum were seen overlying the superficial portion of the bone defect. Bone morphology was more mature and well organized presenting a lamellar pattern compared with bone around defect. Higher magnification of the bone tissue around the block showed that no gaps were present at the bone-biomaterial interface, and the bone seemed to always be in close contact with the block (Figure 6(b)). In some fields, osteoblasts were observed in the process of apposing bone directly on the block surface. No acute inflammatory cells infiltrate, or foreign body reactions were present around the particles or at the interface with the bone. All defects were filled with newly formed dense bone and bone marrow. The tissues present in the defects were composed of 17 ± 4% of BPB/BMSSCs, by 42 ± 2% of the lamellar bone, 12 ± 1% of the woven bone, and by 22 ± 3% of the marrow spaces.

### 3.4. Statistical Evaluation

**Figure 7:**
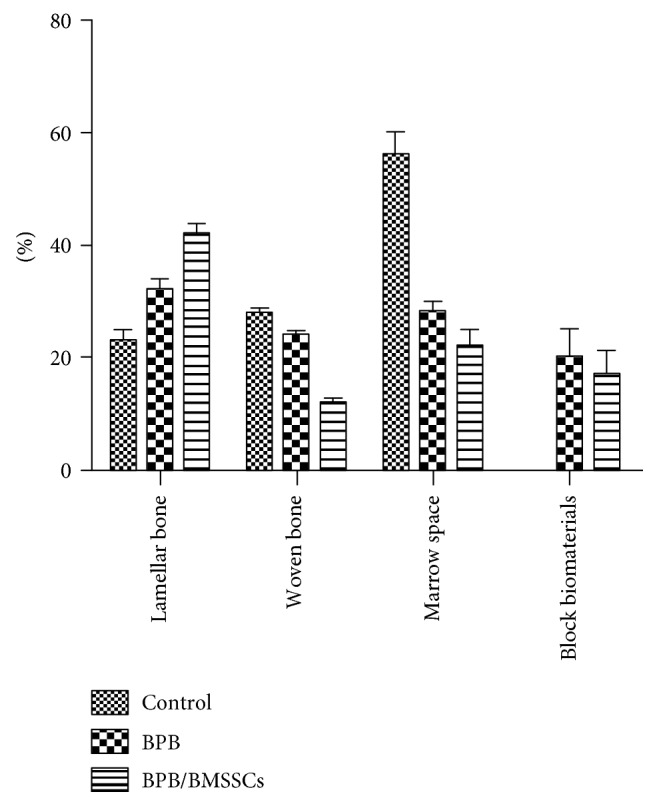
Percentage bar graphic showing difference between site compositions in three groups: control, bone porcine block, and bone marrow stromal stem cells/bone porcine block.

Statistically significant differences were found in the percentage of the marrow space and lamellar bone between the three groups (*P* < .001). In detail, between BPB and BMSSCs/BPB, the difference of the lamellar and woven bones was found to be statistically significant with *P* < .001 and *P* < .01 considering marrow spaces, while no difference was found in biomaterial percentage between the two test groups (Figure 7).

## 4. Discussion

This study found a relevant difference in bone regeneration using a bone porcine block scaffold seeded with bone marrow stromal stem cells when compared with bone porcine block without any kind of treatment. Literature presents a variety of enriching stem/progenitor cells for use in cell-based bone regeneration. They have been explored both clinically and experimentally with varying degrees of success. Bone porcine block scaffolds are promising for a variety of tissue engineering applications. BPB scaffolds have a hard structure which prevents defects and creates the correct environment for bone regeneration without the risk of soft tissue colonization. BPB scaffolds are perfect material for local bone regeneration due to their biocompatibility, osteoconductivity [30], and porosity which allows for efficient BMSSC seeding and growth [31]. BPB biodegradation analysis was not the aim of this study, so the relationship between scaffold biodegradation and bone formation has not been studied in detail, only measured as a percentage in histological evaluations.

In the present study, a 3D software analysis of CBCT was performed and histological images were acquired in which BPB implants were well positioned and induced a higher amount of BMD and SUV within the mandibular defects as compared to BPB without BMSSCs. Despite the contribution of BMSSCs, bone formation is visible in both groups. This study aimed to further characterize bone regeneration induced by BMSSCs in these scaffold-based implants by using histological parameters. The percentages of residual biomaterial, new bone formation, and marrow spaces in the mandibular defects filled with BPB alone or in combination with BMSSCs were evaluated. Histomorphometry was carried out individually for bone, marrow spaces, and residual graft material with a measure error, and the sum will never give 100. Moreover, a qualitative description of the histomorphology of the mandibular defects was provided. The description included the characterization of newly formed bone, the presence of inflammatory cell infiltrate, and the position of bone formation within the defects. The histomorphometric results showed that, at the observed time, the amount of marrow spaces and residual graft material more than new bone formation are always quite different from 100%, this is due to the fact that the three measurements are carried out individually with a margin of error and then the sum of the error is inclusive of the measurement performed in the BPB, lamellar bone, or woven bone and marrow spaces. The results showed that although the percentage of residual biomaterial was similar among the experimental groups, the percentage of formation of new bone and marrow space was different. The present study showed that the BPB/BMSSC group resulted in a significantly greater bone formation in alveolar bone defects in the minipig mandible than BPB groups alone. This suggests that BPB can produce synergic effect with BMSSCs. The minipig was chosen as an animal model because of its similarities to humans in terms of platelet count, clotting parameters, metabolic rate, bone structure, and characteristics of their MSCs [32]. An alveolar defect model offers several advantages for the histologic evaluation of bone tissue-engineered constructs. The surgical procedure is simple, with a limited risk of infection, and a similar intervention by grafting is advocated clinically. A true critical-sized mandibular defect in the minipig model is more than 5 mm [33]. Recently, the synergic effect of MSCs and platelet-rich plasma incorporated into a scaffold on fluorohydroxyapatite and bone formation in surgical bone defects in the edentulous mandible of minipigs has been reported [34]. These results suggest that MSCs show a positive effect on bone formation when combined with autologous growth factors [34]. In fact, the platelet growth factors and biomaterial combination were used with success for bone regeneration [35] and soft tissue augmentation [36, 37]. In the present study, a mandibular defect model was chosen to determine the potential ability of BPB to enhance bone formation when it was supplemented by BMSSCs. Each minipig served as its own control. In our study, BPB alone was used as a scaffold material for testing BMSSC bone induction. The reason for selecting this particular biomaterial was that BPB has been proven to be a suitable carrier for osteoblast-like cells, bone morphogenic proteins, and growth factors. This particular biomaterial has been proven to be a suitable carrier for osteoblast in vertical ridge augmentation of atrophic posterior mandible [38]. This biomaterial has a hard consistency and can be used to block the fragments of the osteotomized segments. When BPB was placed in the surgically created defects, it became properly integrated in the newly lamellar bone during healing [39]. This indicates that the material was osteoconductive and acted as a natural scaffold for new bone formation. Biocompatibility of BPB was confirmed by histological analysis [40]. The formation of a fibrous capsule around the particles did not occur in any section. In this respect, the current findings are consistent with observations made in human histologic studies. These studies indicate that an intimate contact is always established between BPB particles and newly formed mineralized bone [40]. The present findings show that the superior contribution of BMSSCs to bone regeneration is due to the formation of mature (lamellar) bone. Moreover, the contribution of stem cells is evident when analyzing the percentage of woven bone. Qualitative histomorphometry also showed that seeding of stem cells into the scaffolds resulted in greater bone formation in BPB/BMSSCs construct. This is evidenced by the presence of bone tissue in the periphery and in the central area of the defect. Most notably, osteogenesis was observed in the absence of inflammatory processes which may interfere with the process of regeneration [41].

## 5. Conclusion

This study demonstrates that BPB when used as a scaffold to induce bone regeneration may benefit from the addition of BMSSCs in the tissue-engineered constructs. Our data shows the healing pattern in a minipig model, but further research is needed for human applications.

## Abbreviations

BMMNC: Bone marrow mononuclear cell
BMSSC: Bone marrow stromal stem cell
BPB: Bone porcine block.

## Additional Points

*Research Highlights*. Bone marrow stromal stem cells (BMSSCs) were obtained from 6 minipigs. BMSSCs are suitable to be seeded and cultured on a biomaterial such as bone porcine block (BPB). BMSSCs on BPB showed better results in bone regeneration than those on the control and BPB alone groups.
